# Risk and protective factors of near-lethal suicide attempts in adolescents

**DOI:** 10.3389/fpsyt.2026.1744848

**Published:** 2026-03-13

**Authors:** Andrea Chen, Steffen Franke, Abel Stolz, Rainer Papsdorf, Georg von Polier, Daniel Radeloff

**Affiliations:** 1 Clinic for Child and Adolescent Psychiatry, Psychotherapy and Psychosomatics, Medical Faculty, University Leipzig, Leipzig, Germany; 2 Data Integrations Center, University Leipzig, Leipzig, Germany; 3 Institute of Neuroscience and Medicine Brain and Behaviour, Forschungszentrum Jülich, Jülich, Germany; 4 Clinic for Child and Adolescent Psychiatry, Psychosomatics and Psychotherapy, Rheinisch-Westfälische Technische Hochschule (RWTH) Aachen University, Aachen, Germany

**Keywords:** adolescence, attempt, communication, near-lethal, prevention, risk factors, suicide

## Abstract

**Introduction:**

Adolescents exhibit high rates of suicide attempts but comparatively low rates of suicides. While many studies focus on suicide attempts, it is unclear if findings apply to suicides. Studying near-lethal suicide attempts (NLSA) as approximations of suicides is an underexplored approach. This study aimed to identify differences between lower-risk suicide attempts (LRSA) and NLSA.

**Methods:**

Using a standardized clinical survey, suicidal phenomena, risk and protective factors were assessed in a clinical population at first contact from 2018 to 2022. NLSA (N = 17) were identified using strict criteria and compared with LRSA (N = 106) in terms of risk and protective factors using χ² and t-tests for independent samples.

**Results:**

Prior suicide attempts were less frequent in NLSA than in LRSA (58.8% *vs* 84.0%; χ²(1)=5.895, p=.015). This suggests that a history of suicide attempts as an explicit warning sign is less often present in patients with NLSA. Adolescents with NLSA also showed lower levels of both passive and active communication: 73.3% had never been approached by others about suicidality (LRSA: 14.1%; U = 243.500, p <.001) and 60% had never actively disclosed suicidality themselves (LRSA: 28.4%, U = 458.500, p = .022).

**Conclusion:**

The findings highlight the role of communication in suicide prevention, underscoring the need for easily accessible mental health services. Given the small NLSA sample, the results should be interpreted as exploratory and require replication in larger, prospectively designed studies to inform more targeted prevention strategies.

## Introduction

Suicide is a leading cause of death in young people. Globally, it ranks fourth among 15-19-year-olds, with roughly 40,000 deaths each year (1). In Germany, 486 adolescents aged 10–24 died by suicide in 2023. Within this age range, suicide was the leading cause of death (~18% of all deaths), ahead of traffic injuries and malignant neoplasms (1, 2). Although suicide attempts are most prevalent in adolescence and early adulthood (3), suicide mortality in this age group remains comparatively low.

Danish registry data indicate a markedly high attempts-to-suicide ratio in adolescence (156:1 in females and 25:1 in males), which is likely underestimated due to substantial underreporting of suicide attempts (4). These figures highlight the challenge of identifying adolescents at imminent risk of suicide.

Research on suicidal behavior in this age group has primarily followed two approaches. Studies on suicide attempts offer rich clinical data but are limited in their applicability to suicide deaths given the high attempts-to-suicide ratio (4). Studies on suicides rely mainly on large registry datasets, which are necessary due to low incidence rates but often lack detailed psychiatric information; psychological autopsy studies partially address this gap through postmortem interviews with the bereaved (5).

An alternative third approach focuses on near-lethal suicide attempts (NLSA) to combine advantages of studies on suicide attempts and suicides (6). Although NLSA are not uniformly defined across studies, they consistently describe a subgroup characterized by high medical severity (7, 8). Accordingly, NLSA constitute a clinical group that occurs more frequently than suicides, generates systematic healthcare contact data, and shares comparable risk factor profiles with suicide, thereby providing a useful approximation (6, 9, 10). Research settings are heterogeneous (prison, inpatient, emergency, outpatient), and both the terminology and the underlying definitions of NLSA vary across studies, including “violent suicide attempt” (11), “medically serious suicide attempt” (7, 8), “medically severe suicide attempt” (12), “near-fatal suicide attempt” (8, 12), “serious suicide attempt” (13), and “near-lethal suicide attempt” (6, 9, 10).

Several studies suggest that adolescents with NLSA show more pronounced communication difficulties than those with lower-risk suicide attempts (LRSA), including reduced self-disclosure and a diminished ability to articulate psychological pain (7, 8, 12). However, research on NLSA remains limited, particularly in adolescent populations (14–16).

Suicidal behavior is best understood within a hierarchical-ecological framework in which risk and protective factors interact across multiple levels and over time. Distal vulnerabilities include early-life adversity such as maltreatment, enduring personality and coping styles (17–19). Intermediate risk factors comprise clinical and psychosocial factors such as psychiatric disorders, substance use, prior suicide attempts, delinquency, socio-economic adversity and maladaptive stress-coping strategies, including non-suicidal self-injury (NSSI) (11, 18, 20–29). Proximal precipitants involve acute psychosocial stressors (18, 21, 30).

Protective factors similarly operate at individual, relational, and service levels (e.g., adaptive coping and communication skills, social support and integration, and timely access to mental-health care) (15, 18, 24). This multilevel perspective underscores suicide risk as a dynamic process rather than the result of isolated risk or protective factors (18).

Gender differences shape adolescent suicidal behavior: females more often report suicide attempts, whereas males show higher lethality and greater use of high-lethality methods (5, 9, 10). These patterns align with broader phenotypic trends in suicide epidemiology and should be regarded alongside co-occurring risk factors. The present study aimed to compare adolescents with near-lethal suicide attempts (NLSA) to those with lower-risk suicide attempts (LRSA) to identify differences in risk and protective factors as well as psychiatric diagnoses. By examining these differences, we aimed to evaluate whether NLSA can serve as valid proxies for adolescent suicides and to guide targeted suicide prevention strategies.

With this study, we address the following hypotheses:

- The gender ratio in the near-lethal suicide attempt (NLSA) group shows a higher proportion of males compared to the lower-risk suicide attempt (LRSA) group, supporting its role as an approximation of suicide.
- There are differences in ICD-10 diagnoses between groups, with NLSA being associated with affective (F3) and personality disorders (F6).
- NLSA and LRSA differ in the frequency of individual and family risk factors.
- Compared with LRSA, NLSA patients are less likely to communicate suicidality with others, highlighting differences in protective factors like communication behavior.

## Methods

### Data acquisition and sample

This retrospective cohort study is based on a standardized anamnesis form used by mental health professionals at the Department of Child and Adolescent Psychiatry, Psychotherapy and Psychosomatics, University Hospital Leipzig, in routine clinical practice between 2018 and 2022 (NLSA cases were collected between 2018 and 2022). The cohort included all patients up to 18 years of age who presented during the study period. At first contact, a physician or clinical psychologist completed the form in a one-to-one interview; assessments were clinician-rated and supervised by a child and adolescent psychiatrist. If the form was completed more than once, only the first document was used for this study. This establishes the initial contact as the reference point for subsequent analysis. This form is utilized to document suicidal phenomena as well as 16 risk and 5 protective factors at first contact to psychiatry, while lethality was defined by the most severe suicide attempt documented in the clinical record.

The information from the form and other medical context data such as diagnoses, age and gender, were pseudonymized and provided by the Data Integration Center (DIZ) at Leipzig University Hospital. The DIZ is funded by the German Federal Ministry of Education and Research (Grant No. 01ZZ803D) to support patient data services.

The initial sample included all patients for whom the anamnesis form was completed between 2018 and 2022 and consisted of N_0_ = 591 patients. 82 incomplete or inconsistent data sets were excluded. The data sets were classified according to the suicide phenomena (no suicidality n_1_ = 103; suicidal ideation n_2_ = 283; suicide attempts n_3_ = 123).

### Variables and measures

#### Risk factors

The risk factors were measured on a nominal scale (yes/no/not assessed): history of suicide attempts, mental illness with considerable subjective suffering, use of illegal drugs or substance dependence, physical or sexual abuse, impulsiveness, “social drifting” (including homelessness, dependence or delinquency), fantasy of hard suicide methods (imagining or considering highly lethal means of suicide such as hanging or use of firearms), imperative auditory (pseudo-)hallucinations, hopeless or agitated patient, non-suicidal self-injury (NSSI, deliberate self-inflicted harm without suicidal intent, resulting in severe physical damage, e.g. gaping cuts and burns), pain tolerance or habituation, severe physical conflicts and severe or chronic pain due to a somatic illness. Family-related risk factors included suicide attempts or suicides by a family member, mental illness of a family member and use of illegal drugs or substance dependence by a family member. In this study we excluded the risk factor “severe or chronic pain due to a somatic illness” was excluded because, in several NLSA cases, pain resulted from injuries sustained during the suicide attempt rather than a pre-existing somatic condition.

#### Protective factors

For protective factors, the following variables were measured on a nominal scale: Internet search for information on suicidality, internet search for help and internet search for suicide methods or like-minded individuals. Such searches may indicate help-seeking or a need for social connection rather than planning behavior. The communication and help seeking behavior was assessed on an ordinal scale and distinguished between active communication (the adolescent actively approached others to seek help) and passive communication (the adolescent was approached by others). Active communication had the following values: (1) not assessed, (2) patient has never approached anyone about (own) suicidality, (3) has approached underage peers about suicidality, (4) has approached a trusted adult about suicidality, (5) has approached a guardian about suicidality and (6) autonomous request for help at the clinic. Passive communication was measured using the values: (1) not assessed, (2) patient has never talked about (own) suicidality with anyone, (3) has talked about suicidality with underage peers, (4) has talked about suicidality with a trusted adult and (5) has talked about suicidality with a guardian. See **Supplementary Document (S1 Anamnesis Form)** for details.

#### Psychiatric diagnoses

Primary psychiatric diagnoses were extracted from the medical record and coded at the ICD-10 F-chapter level (F1-F9). Primary diagnoses were assigned by child and adolescent psychiatrists based on clinical presentation, observation, and psychodiagnostic procedures. Given the association between personality disorders and self-harming behavior, the presence of a primary or secondary diagnosis within this diagnostic category was included in the analysis as a binary indicator. F6-diagnoses were made in accordance with the German-language AWMF guideline for the diagnosis and treatment of Emotionally unstable personality disorder, which explicitly endorses diagnosis before the age of 18 when diagnostic criteria are fulfilled in order to enable timely, developmentally appropriate intervention (31). Given the small NLSA group and heterogeneous primary diagnoses, ICD-10 diagnoses were aggregated to F-chapters to avoid sparse categories and to ensure interpretable comparisons.

#### Definition of LRSA and NLSA

According to Bridge et al. (2006) (21), a suicide attempt is defined as “a non-fatal, self-inflicted destructive act with explicit or inferred intent to die”. In line with this definition, non-suicidal self-injury (NSSI) without suicidal intent was excluded. Suicide attempts were categorized into lower-risk suicide attempts (LRSA, n3.2 = 106, 86.18%) and near-lethal suicide attempts (NLSA, n3.1 = 17, 13.82%) based on the most severe suicide attempt since initial contact with the Department of Child and Adolescent Psychiatry. The present study focused on comparing the medical lethality of suicide attempts rather than method classification. A near-lethal suicide attempt was defined as an act that was (1) potentially fatal and (2) characterized by loss of control over survival, such that survival depended on external factors, including chance discovery and/or rapid and effective emergency intervention. For intoxication, near-lethality was defined as ingestion of a potentially lethal dose (LD50) without prior disclosure to a third party. Strangulation met near-lethal criteria when it resulted in loss of consciousness, and jumping when it involved a minimum height of five meters. No cases involving traffic–related suicide attempts were identified. LRSA were defined as attempts not fulfilling NLSA criteria and were characterized by lower medical severity or maintained control over survival (e.g., sublethal intoxications with timely disclosure, superficial cutting, or low-height jumps without serious injury). NLSA cases are shown in **Table 1**.

**Table 1:** Suicide methods of patients with near-lethal suicide attempts.

Suicide method	Total	C	A	m	f
Intoxicaton	6	2	4	0	6
Jumping	9	0	9	4	5
(Attempted) hanging	2	2	0	2	0

C, child (≤ 13 years); A, adolescent (14-18 years); m, male; f, female.

### Statistical methods

Statistical analyses were focused on NLSA and LRSA. χ²- and an independent samples t-test was performed to examine potential disparities between the two groups in terms of demographic characteristics, such as age and gender. The primary psychiatric diagnoses were classified and simplified according to the primary ICD-10 F chapters (e.g. F1, F2, F3). An additional variable was created to indicate the presence of an F6 diagnosis (Disorders of adult personality and behavior), whether as a primary or secondary diagnosis.

Nominal variables were compared using χ² tests or Fisher’s exact test, depending on the sample size. Ordinal variables were compared using Mann-Whitney U tests. Odd’s ratios were calculated to determine the relative risk between the groups. All statistical tests were performed with α = 0.05 and p-values between 0.05 and 0.10 were interpreted as indicating a statistical trend.

### Software and resources

SPSS (Version 29.0.0.0) was used to perform statistical analyses. The AI translator DeepL (Version 24.11.31463097) was used to assist the wording of the manuscript. GraphPad Prism (Version 10.4.1) was used to create the figures.

### Ethics

The study was approved by the Ethics Committee of the Medical Faculty of the University Hospital Leipzig, Germany (study ID: 199/22-ek) and was conducted in accordance with the Declaration of Helsinki. As a retrospective analysis of routinely collected, pseudonymized clinical data, informed written consent was not required.

## Results

### Sample and descriptive statistics

Of all individuals with a suicide attempt, 107 were female (87.00%) and 16 were male (13.00%) resulting in a gender ratio of 6.7. Age ranged from 7.9 to 17.9 years, with a mean of 15.4 years (SD = 1.85).

The gender ratio differed between the NLSA and LRSA group (χ^2^(1) = 4.691; Fisher’s exact test p = .046). In the LRSA group, 10.4% of the subjects were male and 89.6% were female (1:8.6), while in the NLSA group, 29.4% of the subjects were male and 70.6% were female (1:2.4). Age did not differ between the groups (t(121) = .414, p = .679).

### Group comparisons

#### Risk factors

Two of the 15 risk factors analyzed showed differences between the groups: “history of suicide attempts” (χ^2^(1) = 5.895, Fisher’s exact test p = .023, OR = 0.273 [0.091, 0.817]) and “fantasy of hard suicide methods” (χ2(1) = 3.894, p = .048, OR = 3.556 [0.946, 13.357]).

**Figure 1:**
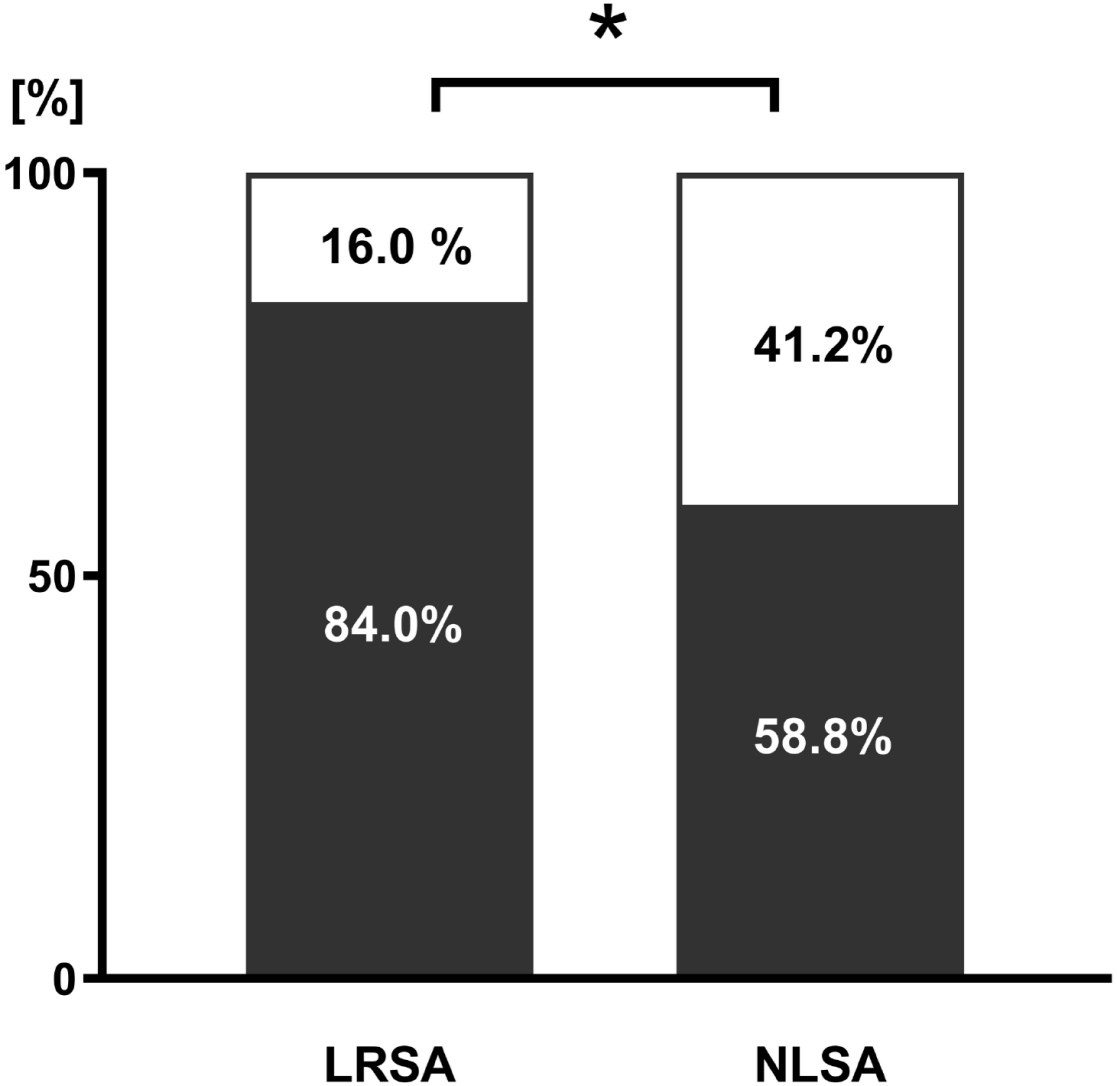
History of suicide attempts. LRSA, lower risk suicide attempts; NLSA, near-lethal suicide attempt; black = Percentage of patients reporting a previous suicide attempt, white = Percentage of patients reporting no previous suicide attempts. * = p < 0.05.

In the NLSA group, only 58.8% reported a history of suicide attempts compared to 84.0% in the LRSA group at first contact with the department of child and adolescent psychiatry (**Figure 1**). Moreover, 80.0% of NLSA patients indicated fantasizing about hard suicide methods prior to the attempt, compared to 52.9% in the LRSA group.

#### Protective factors

**Figure 2:**
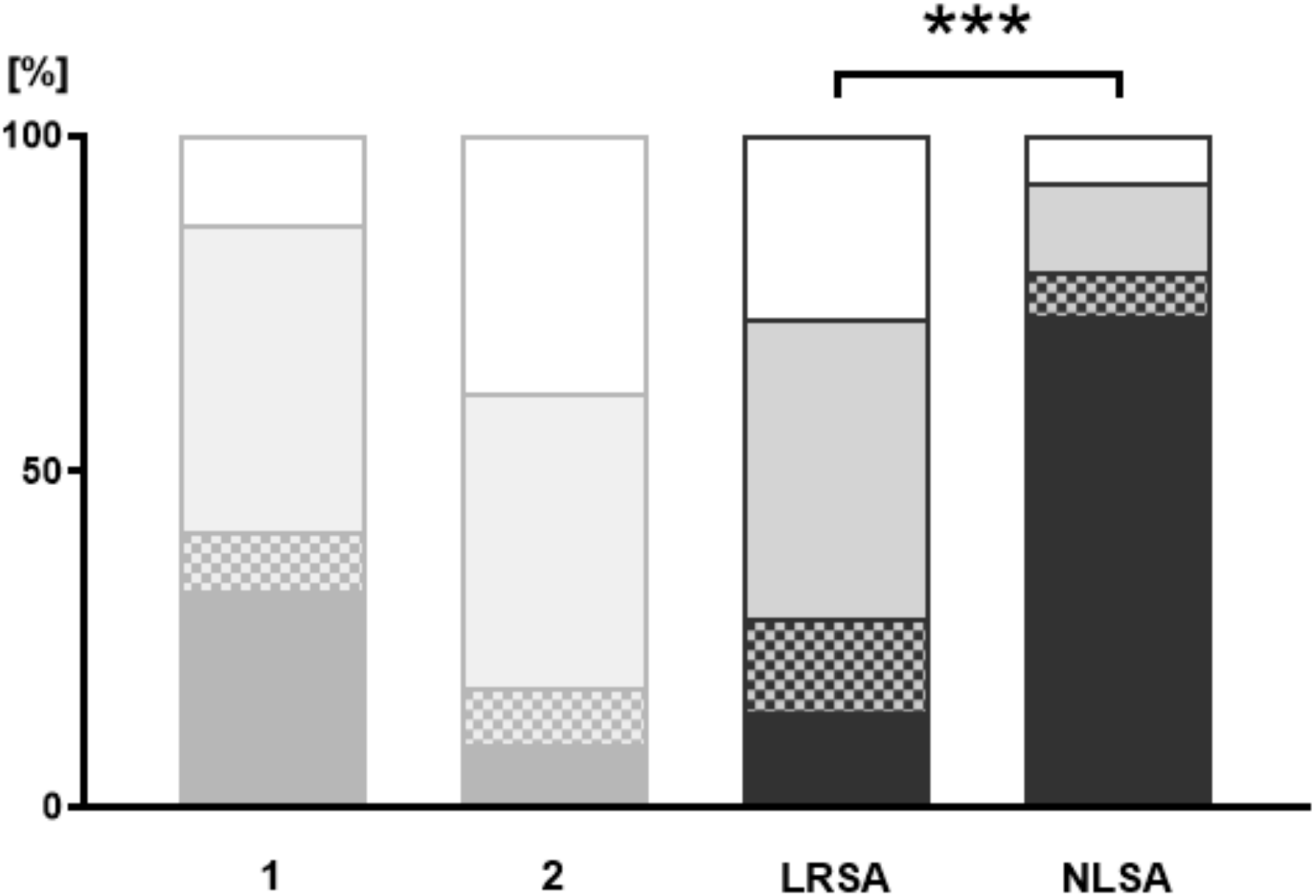
Passive communication. Illustration of the patients’ responses to the topic: Who has ever approached you about suicidality? The NLSA and LRSA groups are compared; additional data from groups “no suicidal ideation” (1) and “suicidal ideation” (2) are shown for better illustration (shaded in grey). Pattern coding: Black = “patient has never been approached by anyone about (own) suicidality”; tile pattern = “has been approached by underage peers about suicidality”; grey = “has been approached by a trusted adult about suicidality”; white = “has been approached by a guardian about suicidality”; dotted white = has been approached by a guardian about suicidality. *** = p < 0.001.

According to the data, adolescents with NLSA were less frequently (ever) approached about suicidality before the suicide attempt (U = 243.500, p <.001). While 14.1% of the LRSA patients were not approached about suicidality, this applied to 73.3% of the NLSA patients (**Figure 2**). Moreover, differences were found in active communication about suicidality (U = 458.500, p = .022).

**Figure 3:**
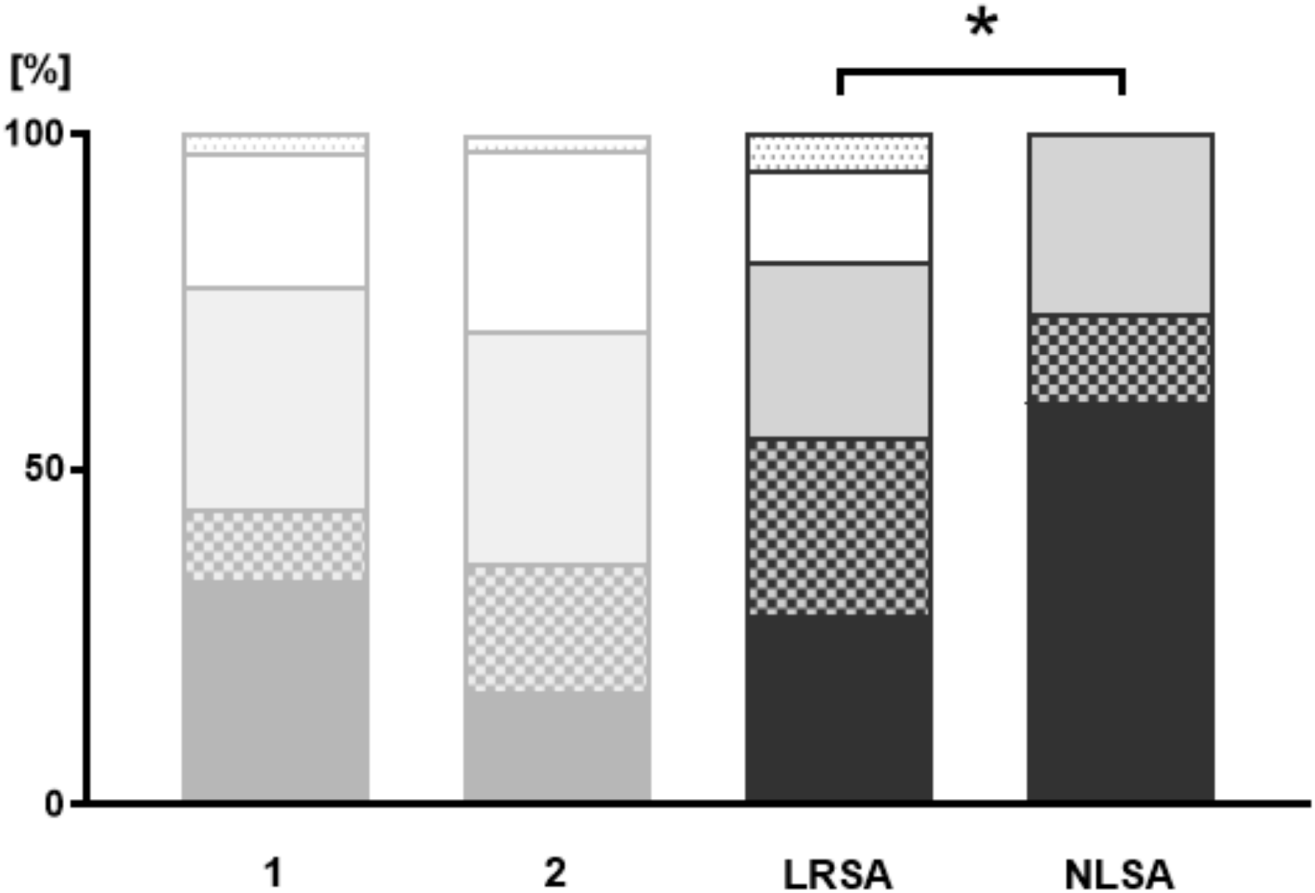
Active communication. Illustration of the patients’ responses to the question: Who did you talk to about suicidality? The NLSA and LRSA groups are compared; additional data from groups “no suicidal ideation” (1) and “suicidal ideation” (2) are shown for better illustration (shaded in grey). Pattern coding: black = “patient has never approached anyone about (own) suicidality”, tile pattern = “has approached underage peers about suicidality”, grey = “has approached a trusted adult about suicidality”, white = “has approached a guardian about suicidality”, dotted white = “autonomous request for help at the clinic”. * = p < 0.05.

In the LRSA group, 28.4% of the patients reported that they have never approached anyone about suicidality, compared to 60.0% in the NLSA group (**Figure 3**). Conversations about suicidality with underage peers were initiated by 26.3% of LRSA and 13.3% of NLSA patients. Of the LRSA group, 26.3% of the patients have approached trusted adults about suicidality and 26.7% of NLSA patients. Conversations with guardians were initiated in 13.7% of the LRSA and 0.0% in the NLSA group. Autonomous requests for help at the clinic were indicated by 5.3% of LRSA patients.

Regarding passive communication, 14.1% of LRSA and 73.3% of NLSA patients reported that they have never been approached by anyone about suicidality. In 14.1% of LRSA and 6.7% of NLSA patients, conversations about suicidality were initiated by their underage peers. In the LRSA group, 44.7% were approached about suicidality by a trusted adult, compared to 13.3% of the NLSA group. Conversations about suicidality initiated by their guardians were reported by 27.1% of LRSA and 6.7% of NLSA patients. For details, see **Table 2**.

**Table 2:** Comparison of influencing factors on suicidality between LRSA and NLSA groups in adolescent patients.

Variable	n	Test	Value	p	Sign. level	OR Value	OR [95% CI]
Risk factor							
HSA	123	F	5.895	0.023	*	0.273	[0.091; 0.817]
MI	122	F	0.237	1.000	n.s.	1.684	[0,202; 14,070]
DU	123	F	0.063	1.000	n.s.	0.857	[0,258; 2,849]
PSA	103	F	1.531	0.335	n.s.	0.382	[0,080; 1,839]
I	119	χ²	1.936	0.164	n.s.	2.500	[0,664; 9,409]
SD	117	F	0.583	0.430	n.s.	1.712	[0,425; 6,889]
FHSM	117	χ²	3.894	0.048	*	3.556	[0.946; 13,357]
IV	115	F	0.185	0.650	n.s.	1.429	[0,279; 7,309]
H	118	χ²	2.320	0.128	n.s.	2.259	[0,777; 6,565]
F-HSA	83	F	0.247	0.749	n.s.	1.364	[0,400; 4,647]
F-MI	97	F	2.641	0.174	n.s.	0.371	[0,109; 1,267]
F-DU	121	F	0.284	0.758	n.s.	0.696	[0,183; 2,651]
NSSI	119	F	0.846	0.472	n.s.	1.785	[0,513; 6,213]
PT	117	F	0.148	0.766	n.s.	1.250	[0,401; 3,898]
PA	118	F	0.958	0.296	n.s.	2.286	[0,419; 12,458]
F3	123	χ²	0.010	0.921	n.s.	1.054	[0.373; 2.981]
F6	123	F	4.028	0.080	n.s.	4.329	[0.931; 20,123]
Protective factor							
AC	110	U	458.500	0.022	*	a	a
PC	100	U	243.500	< 0.001	***	a	a
IS-IS	82	F	0.027	1.000	n.s.	0.895	[0,238; 3,360]
IS-H	76	F	2.722	0.191	n.s.	b	b
IS-SM	71	F	0.027	1.000	n.s.	1.132	[0,256; 5,008]

HSA, History of suicide attempt; MI, Mental illness; DU, Drug use; PSA, Physical or sexual abuse; I, Impulsiveness; SD, Social drifting; FHSM, Fantasy of hard suicide methods; IV, Imperative voices; H, Hopelessness; F-HSA, Family: History of suicide attempt or suicide; F-MI, Family: Mental illness; F-DU, Family: Drug use; NSSI, Nonsuicidal self-injury; PT, Pain tolerance; PA, Physical altercations; F3, F3 primary diagnosis; F6 ,F6 diagnosis; AC, Active communication; PC, Passive communication; IS-IS, Internet search for information on suicidality; IS-H, Internet search for help; IS-SM, Internet search for suicide methods; F, Fisher exact test; χ² , χ²-Test; U, Mann-Whitney U Test; n.s. ,not significant; *p < 0.05; ***p < 0.001; a: Value was not determined because variable is not binary; b: Value was not determined because the frequency of one field was zero.

#### Psychiatric disorders

The analysis of the ICD-10 diagnoses revealed no group differences for the primary F chapters, e.g. F3 (χ²(1) = .010, p = .921, OR = 1.054 [0.373, 2.981]). For F6 diagnoses, 5 LRSA patients were diagnosed with a personality disorder as a primary or secondary diagnosis and 8 patients within the NLSA-group. A trend was observed suggesting a potential association with NLSA (χ²(1) = 4.028, Fisher’s exact test p = .080, OR = 4.329 [0.931, 20,123]).

## Discussion

The aim of our study was to compare influencing factors among adolescent suicide attempters to identify characteristics associated with a near-lethal outcome. Given the small size of the NLSA group, the results should be interpreted as exploratory, providing preliminary indications rather than definitive conclusions. Our main findings show the following:

(A) In the NLSA group, the gender ratio was shifted toward males compared to LRSA. (B) The analysis of risk factors revealed that previous suicide attempts were less frequent in individuals of the NLSA group compared with the LRSA group at first contact to psychiatry. NLSA patients are more associated with fantasizing about hard suicide methods (this is likely attributable to the definition of lethal suicide attempts). (C) No differences were found for affective disorders diagnosis (ICD-10 F3), while a trend (p<0.10) suggested a stronger association between personality disorder diagnosis (ICD-10 F6) and NLSA than LRSA. (D) With regard to the protective factors, considerable differences in communication behavior were observed: Compared to LRSA, individuals with NLSA were less likely to be approached about suicidality by third parties (passive communication) and were less likely to actively address their suicidality to others (active communication) at first contact to psychiatry.

Previous research shows a gender pattern in adolescent suicidality, with females more frequently attempting suicide and males exhibiting higher lethality (21, 28, 32). In our data, the higher proportion of males in the NLSA group is best interpreted as reflecting phenotypic similarity to established suicide patterns. Accordingly, gender should be considered in conjunction with co-occurring clinical and social risk factors rather than in isolation.

In adults, a previous suicide attempt emerged as strong risk factor for suicide (22). In adolescents, the role of previous suicide attempts as a predictor of suicide is less consistent in the literature: While many studies suggest that the risk of suicide death increases with repeated attempts (21, 33, 34), individual studies have shown the potentially fatal nature of a first attempt (35–38), particularly when violent methods are used (39–41) and no prior intervention has occurred (36). In a U.S. sample, 71.4% of first suicide attempts among 10-24-year-olds were fatal (36); similarly, Swedish data show that 73% of youths who died by suicide had no documented prior attempt (35). Our results are consistent with this finding, indicating a heightened risk of a near-lethal outcome on the first attempt.

Speculative, adolescents with an NLSA may exhibit reduced ambivalence and more extensive planning of the approach. Increased impulsivity (and thus a reduced inhibition threshold for a hard suicide method) was not found in NLSA compared to LRSA.

Our study results indicate that a high proportion of adolescents with the highest risk of suicide does not exhibit previous suicidal behavior. Moreover, there are indications that adolescents rarely seek psychiatric help before suicide (28). Accordingly, it is crucial for suicide prevention in adolescence that low-threshold support services are expanded.

The item “fantasy of hard methods” indicates the presence of suicidal ideation involving hard methods but has limited operational clarity. Moreover, it is partly circular, as adolescents with NLSA are more likely to have used hard methods and, consequently, to report ideation involving these methods. We therefore report this variable descriptively, analyze it separately, and refrain from substantive interpretation.

It is known from psychological autopsy studies that nearly 90% of adolescents dying by suicide met the criteria for a mental illness (21). However, the vast majority were not in treatment (21). Among adults, affective and personality disorders are among the mental disorders with the highest risk of suicide (22, 42). In adolescents, research has shown that personality (21, 29) and affective disorders, particularly in severe manifestations (16, 21), may increase the lethality of suicide attempts. This is consistent with our study results, which show - as a trend - a stronger association between personality disorder (ICD-10 F6) and NLSA compared to LRSA. However, given ongoing developmental changes in adolescence, interpretation is limited by the diagnostic uncertainty of personality disorders before the age of 18. No associations were found between severity levels of suicide attempts and affective disorders (ICD-10 F3). Aggregating ICD-10 diagnoses to F-chapters limits disorder-specific inferences. Future studies using dimensional, longitudinal designs and larger samples should examine diagnosis-level patterns in greater detail (e.g., within F3).

Our results show that adolescents with NLSA are less likely to communicate with others about their suicidality compared to LRSA. In the NLSA group, 60.0% stated that they had never approached anyone (active communication) about suicidality (LRSA: 28.4%) at first contact with the department of child- and adolescent psychiatry. None of the adolescents in the NLSA group actively approached their parents or the department of child and adolescent psychiatry about their own suicidal intentions (LRSA: 13.7%/5.3%). Similarly, an overwhelming majority of 73.3% in the NLSA group had never been approached by third parties (passive communication) about suicidality (LRSA: 14.1%).

There are various interpretations for this finding. Several mechanisms may lead to this pattern: adolescents with NLSA may show less ambivalence and greater non-disclosure, have limited communication skills and reduced support or communicative initiative from their environment. Additional interacting factors can further reduce the likelihood of passive communication by others: insecure attachment (less trust in others, avoidance) (19, 43), emotion regulation difficulties (high arousal, limited coping mechanisms) (44, 45), stigma that suppresses help-seeking (46, 47) also may inhibit others from addressing suicidality, as well as family dynamics characterized by low warmth or high conflict, resulting in fewer supportive check-ins (30, 48, 49). In combination, these mechanisms may increase the likelihood of higher-lethality attempts with fewer observable warning signs, consistent with our communication findings.

Previous research shows that passive communication appears to be a key protective factor by offering emotional support, encouraging professional help-seeking, and reducing the risk of a fatal outcome (50). In turn, a lack of communication amplifies social isolation and emotional neglect (51, 52), potentially increasing the lethality of suicide attempts (28).

Our findings emphasize the need to actively address possible suicidality in adolescents by their environment. Barriers preventing adolescents from communicating suicidality should be identified and lowered by normalizing conversations and reducing stigma.

The study highlights the need to further develop prevention and treatment programs by addressing factors associated with NSLA. Encouraging early, low-threshold communication, particularly passive contact, could be an easily implemented and effective protective measure. In addition to confidants, first-contact professionals, including teachers, pediatricians, psychiatrists and emergency department teams are especially important for initiating such communication. Early interventions may help identify at-risk adolescents and provide targeted support.

Prevention should operate across multiple levels, with school-based programs to improve detection and intervention (53, 54), proactive screening and brief interventions in pediatric and primary care (55), emergency-department (56, 57) and individual safety planning (58), low-threshold, anonymous access to crisis hotlines and internet-based support (59, 60), and promotion of regular physical activity as a low-threshold complementary strategy are linked to better mental-health outcomes (61, 62).

### Strengths, limitations and future implications

The present study is one of the few studies to examine NLSA as an approximation of suicide in adolescence. This approach provides a high level of clinical detail for the cases analyzed.

The small NLSA sample size reflects the low prevalence of severe suicide attempts despite years of data collection. However, the small number of NLSA highlights the importance of distinguishing between NLSA and LRSA in research in this age group: without this distinction, the generalizability of findings from suicide attempts to suicides would be limited, as LRSA represent the vast majority of events. Moreover, NLSA cases are difficult to identify, as some adolescents at the highest risk may die by suicide rather than survive a near-lethal attempt, further underscoring the close resemblance between NLSA and suicides.

The risk and protective factors were assessed at first clinical contact, while lethality was defined by the most severe suicide attempt documented subsequently. This timing mismatch means that some factors may have changed between assessment and the index event, which can weaken or shift correlations. Findings should therefore be interpreted as links between a baseline profile and subsequent maximum lethality and not as concurrent predictors. Future prospective studies with repeated time points are needed to address this more directly.

## Conclusion

This research examines an understudied phenomenon: adolescents with near-fatal suicide attempts. The study highlights important risk and protective factors and examines differences between NLSA and LRSA. Firstly, the results illustrate the importance of communication as a suicide prevention measure: over 70% of individuals of the NLSA group were not approached about suicidality by their environment. Secondly, about 40% of those in the NLSA group reported that they never had attempted suicide when they first contacted the child and adolescent psychiatry department (compared to 16% of the LRSA). This indicates that patients with NLSA in particular lack certain previous warning signs.

The results emphasize the need to remove barriers to mental health care and promote communication about suicidality. Future research should aim for larger NLSA samples and a prospective design. This will help to better understand the pathways to mental health care and to better target prevention strategies.

## Data Availability

The data analyzed in this study is subject to the following licenses/restrictions: data security aspects. Requests to access these datasets should be directed to andrea.chen@medizin.uni-leipzig.de; daniel.radeloff@medizin.uni-leipzig.de.
