# Supplementary material for: Risk and protective factors of near-lethal suicide attempts in adolescents
Source: Front Psychiatry. 2026 Mar 13;17:1744848. doi: 10.3389/fpsyt.2026.1744848 (PMC13033975; doi:10.3389/fpsyt.2026.1744848)
Supplement: Supplementary file 1 [file DataSheet1.docx]

**Anamnesebogen Suizidalität**

**Anamnesebogen Suizidalität**

| Datum: |
| --- |

**(A) Intensität suizidaler Phänomene**

**aktuell:**

nicht erhoben

**jemals:**

nicht erhoben

**Frequenz**

trifft nicht zu / keine Angaben

**Zeitlicher Verlauf**

trifft nicht zu / keine Angaben

**Suizidgedanken erstmals (Monat/Jahr): XX/XXXX**

**Suizidversuche:**

Anzahl bisheriger Suizidversuche: nicht erhoben

Letzter Suizidversuch (Monat/Jahr): XX/XXXX

Schwerste Suizidhandlung:

Monat/Jahr, Methode, kurze Schilderung

| Erläuterungen: |
| --- |

**(B) Risikofaktoren**

**Patient:** ja nein nicht erhoben

Suizidversuche in der Vorgeschichte

Psychische Erkrankung mit erheblichem subjekt. Leiden

Konsum illegaler Drogen/Substanzabhängigkeit

körperliche Misshandlung / sexueller Missbrauch

Impulsivität

„social drifting“: Obdachlosigkeit/Abgängigkeit/Delinquenz

Phantasie harter Suizidmethoden

imperative Stimmen

hoffnungsloser oder getriebener Patient

**Familie:** ja nein nicht erhoben

Suizidversuche/Suizide

Psychische Erkrankungen

Konsum illegaler Drogen/Substanzabhängigkeit

| Erläuterungen: |
| --- |

**(C) Grenzverletzendes Verhalten** ja nein nicht erhoben

NSSI mit schwerer körperlicher Schädigung

(z.B. klaffende Schnitte, Verbrennungen)

Schmerztoleranz / Habituation

Schwere körperliche Konflikte

Schwerste oder chronische Schmerzen durch somat. Erkrankung

| Erläuterungen: |
| --- |

**(D) Kommunikation und Inanspruchnahme von Hilfe**

Aktive Kontaktaufnahme:

nicht erhoben

passive Kontaktaufnahme:

Wählen Sie das höchstrangige Element aus.

ja nein nicht erhoben

Patient hat sich im Internet über Suizidalität informiert

Suche nach Hilfe im Internet

Suche nach Suizidmethoden oder Gleichgesinnten im Internet

**Beurteilung**

**AiW Einstufung**

**Risikostufe: 01234N**

**OA Einstufung (ab Risikostufe 3)**

**Hochrisikopatient: nicht beurteilt vorläufig beurteilt nein ja**

**Formular ‚Reevaluation‘ nutzen? ja nein**

Erläuterungen:
